# We Thank You, Gentlemen

**Published:** 1893-03

**Authors:** 


### WE THANK YOU, GENTLEMEN.

We have never thought it in very good taste to publish the letters of commendation which we have received from time to time. Just at this juncture, however, we may be excused for departing from our usual custom by printing the following:

McKINNEY, TEXAS, December 21, 1892.

*Atlanta Medical and Surgical Journal, Atlanta, Ga.:*

GENTLEMEN—Enclosed find P. O. order for two ($2) dollars for my subscription to THE JOURNAL. I don't think I speak disparagingly of its previous management when I say I think THE JOURNAL has been filled with better and more instructive literature this year than formerly. Yours truly,

I. E. W.

JANESVILLE, CALIFORNIA, February 4, 1893.

*Atlanta Medical and Surgical Journal:*

GENTLEMEN—Some months ago I received a sample copy of your JOURNAL. I am well pleased with the contents and especially the arrangement of same—the reading matter being in one place and the advertisements in another. I also like your large clear type. Respectfully,

A. H. J.

TROY, S. C., February 13, 1893.

DEAR JOURNAL—I have concluded to continue my subscription to THE ATLANTA MEDICAL AND SURGICAL JOURNAL for another year. The last number is so interesting that I am anxious for more of the same sort,

